# Supplementary material for: Independent mobility of proteins and lipids in the plasma membrane of Escherichia coli
Source: Mol Microbiol. 2014 Apr 30;92(5):1142–53. doi: 10.1111/mmi.12619 (PMC4276291; doi:10.1111/mmi.12619)
Supplement: Supplementary file 1 [file mmi0092-1142-SD1.pdf]

Molecular Microbiology (2014) 92(5), 1142–1153
doi:10.1111/mmi.12619
First published online 30 April 2014

Independent mobility of proteins and lipids in the plasma membrane of *Escherichia coli*

Anja Nenninger,[1] Giulia Mastroianni,[1] Alexander Robson,[2] Tchern Lenn,[1] Quan Xue,[2] Mark C. Leake[3] and Conrad W. Mullineaux[1]*

[1] *School of Biological and Chemical Sciences, Queen Mary University of London, Mile End Road, London E1 4NS, UK.*
[2] *Clarendon Laboratory, Department of Physics, University of Oxford, Parks Road, Oxford OX1 3PU, UK.*
[3] *Biological Physical Sciences Institute (BPSI), Departments of Physics of Biology, University of York, York YO10 5DD, UK.*

Summary

Fluidity is essential for many biological membrane functions. The basis for understanding membrane structure remains the classic Singer-Nicolson model, in which proteins are embedded within a fluid lipid bilayer and able to diffuse laterally within a sea of lipid. Here we report lipid and protein diffusion in the plasma membrane of live cells of the bacterium *Escherichia coli*, using Fluorescence Recovery after Photobleaching (FRAP) and Total Internal Reflection Fluorescence (TIRF) microscopy to measure lateral diffusion coefficients. Lipid and protein mobility within the membrane were probed by visualizing an artificial fluorescent lipid and a simple model membrane protein consisting of a single membrane-spanning alpha-helix with a Green Fluorescent Protein (GFP) tag on the cytoplasmic side. The effective viscosity of the lipid bilayer is strongly temperature-dependent, as indicated by changes in the lipid diffusion coefficient. Surprisingly, the mobility of the model protein was unaffected by changes in the effective viscosity of the bulk lipid, and TIRF microscopy indicates that it clusters in segregated, mobile domains. We suggest that this segregation profoundly influences the physical behaviour of the protein in the membrane, with strong implications for bacterial membrane function and bacterial physiology.

Accepted 14 April, 2014. *For correspondence. E-mail c.mullineaux@qmul.ac.uk; Tel. (+44) 20 7882 3645; Fax (+44) 20 7882 7427.

Introduction

The standard structural description for biological membranes for over 30 years has been the fluid mosaic model of Singer and Nicolson (1972) which envisages membrane-integrated proteins immersed in a 'sea' of lipid. Unless they are immobilized by other interactions (e.g. with cytoskeletal proteins) membrane proteins are free to diffuse among the surrounding lipid molecules. This model has been modified in recent years as a result of numerous studies showing patchy or compartmentalized distributions of specific lipids and proteins in both eukaryotic and prokaryotic cell membranes (reviewed by Vereb *et al*., 2003; Engelman, 2005; Matsumoto *et al*., 2006; Jacobson *et al*., 2007; Mileykovskaya and Dowhan, 2009). Fluorescent protein tagging of various bacterial plasma membrane proteins has revealed their concentration in mobile microdomains (Johnson *et al*., 2004; Lenn *et al*., 2008; López and Kolter, 2010; Llorente-Garcia *et al*., 2014). However, the forces that cause the clustering of specific proteins remain obscure.

Current membrane models suggest that the diffusion of all membrane components must be strongly influenced by the viscosity of the lipid bilayer, which in turn is influenced by temperature and lipid composition (Murata, 1989; Thompson, 1989; Fulbright *et al*., 1997; Jin *et al*., 1999; Szalontai *et al*., 2000; Lindblom *et al*., 2002; Sarcina *et al*., 2003). When most biological membranes are cooled to around 10°C below growth temperature, there is a step decrease in the fluidity of the lipid bilayer, attributed to a phase transition from the fluid liquid crystalline state to the more viscous crystalline gel state, observed by measuring the mobility of reporter molecules embedded in the lipid bilayer (Fulbright *et al*., 1997; Jin *et al*., 1999; Lindblom *et al*., 2002; Sarcina *et al*., 2003). Cooling below phase transition has pronounced effects on the translational and rotational diffusion of lipids, and on the flexing of the fatty acid tails (Szalontai *et al*., 2000; Lindblom *et al*., 2002; Denich *et al*., 2003; Sarcina *et al*., 2003). Bacteria such as *Escherichia coli* alter their membrane lipid composition in response to environmental and physiological stress to maintain a constant membrane viscosity by 'homeoviscous adaptation' (Morein *et al*., 1996; Rilfors and Lindblom, 2002).

© 2014 The Authors. *Molecular Microbiology* published by John Wiley & Sons Ltd.
This is an open access article under the terms of the Creative Commons Attribution License, which permits use, distribution and reproduction in any medium, provided the original work is properly cited.

It has been widely assumed that lipid phase transition must also affect the mobility of membrane proteins. However, there is surprisingly little direct evidence that lipid phase transition affects membrane protein mobility. In a classic early experiment (Frye and Edidin, 1970), the mixing of cell surface antigens in fused cultured mammalian cells is retarded at low temperatures, and this is attributed to slower membrane protein diffusion due to greater lipid viscosity. More recent studies using more direct measuring techniques have shown that rotational (Aisenbrey and Bechinger, 2004) and translational (Tominaga *et al*., 2004) diffusion of proteins in reconstituted model membranes is slower below the lipid phase transition temperature. However real biological membranes have more complicated mixtures of lipids and proteins, and it cannot be assumed that their behaviour is the same.

In a study of the dynamic properties of the cytoplasm of *E. coli,* we used a series of fluorescent protein constructs expressed in the cell to determine the size-dependence of cytoplasmic diffusion. The behaviour of the constructs was simpler and more predictable than that of indigenous proteins; clearly the behaviour of the indigenous proteins was complicated by interactions with partners in the cell (Nenninger *et al*., 2010). In the present study we have applied a comparable approach to study the 'generic' behaviour of membrane proteins by expressing in *E. coli* cells a simple, non-native membrane protein with a GFP tag. We used confocal Fluorescence Recovery after Photobleaching (FRAP) and video-rate Total Internal Reflection Fluorescence (TIRF) microscopy to examine the distribution and mobility of this protein as a function of temperature. For comparison, we carried out similar measurements on a fluorescent lipid probe and a well-characterized GFP-tagged native membrane protein.

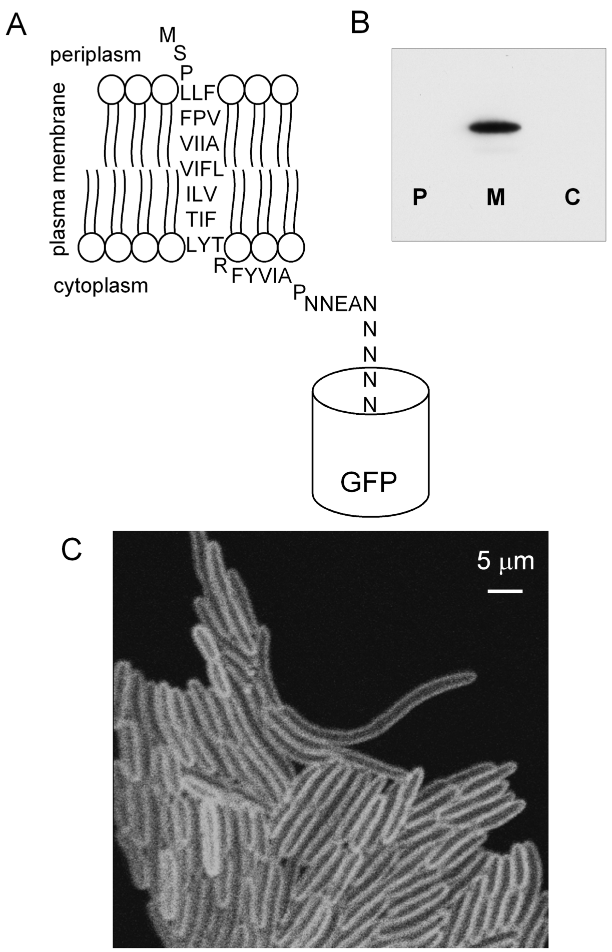

Fig. 1. The helix1021-GFP protein construct and its location in the cell.
A. Predicted membrane topology of the construct.
B. Western blot with anti-GFP antibody blotted against periplasmic (P) plasma membrane (M) and cytoplasmic (C) fractions isolated from a culture of *E. coli* expressing helix1021-GFP.
C. Laser scanning confocal fluorescence micrograph showing GFP fluorescence from *E. coli* cells expressing helix1021-GFP.

Results

Expression of a model membrane protein in E. coli

We constructed a gene coding for a model membrane protein using as a starting point a truncated version of open reading frame *sll1021* from the cyanobacterium *Synechocystis* sp. PCC6803, coding for a membrane protein with no identified homologues in *E. coli*. The protein has unknown function but has been identified in the plasma membrane of *Synechocystis* (Huang *et al*., 2002). The predicted gene product has 673 amino acids with a single predicted transmembrane alpha-helix close to the N-terminus. The ends of the predicted transmembrane helix lack any of the two-residue motifs that appear important for specific interactions with phospholipid head-groups (Hunte, 2005). We took a portion of the *sll1021* sequence coding for 38 amino acids including the predicted transmembrane alpha-helix and fused it in-frame to the gene coding for GFPmut3* (Cormack *et al*., 1996) with a linker of five asparagine residues. The construct was expressed in *E. coli* cells from the arabinose-inducible pBAD24 vector (Guzman *et al*., 1995). The predicted topology of the model protein (helix1021-GFP) is illustrated in Fig. 1A. The GFP domain is almost certainly on the cytoplasmic side of the membrane, since there are no Sec or Tat leader sequences present to initiate the translocation of GFP into the periplasm (Thomas *et al*., 2001; Natale *et al*., 2008). Cells expressing helix1021-GFP were fractionated and proteins of the periplasmic, cytoplasmic and plasma membrane fractions separated by SDS-PAGE and probed with anti-GFP antibody. GFP was only detected in the plasma membrane fraction, where the antibody recognized a

protein migrating at 28–29 kDa, consistent with the predicted gene product (Fig. 1B). The band ran at a slightly higher molecular weight than free GFP, confirming that the protein integrates into the plasma membrane as expected, and the GFP tag is not cleaved off the transmembrane helix. Confocal fluorescence micrographs of GFP fluorescence from cells expressing the protein are consistent with membrane localization. Images recorded at high *z*-axis resolution show green fluorescence confined to a halo at the periphery of the cell (Fig. 1C), a characteristic of confocal images of GFP-tagged membrane proteins in *E. coli* (Mullineaux *et al*., 2006).

Localization of the fluorescent lipid probe BODIPY FL-C_{12} in E. coli *cells*

For comparison with the behaviour of our model membrane protein we used an artificial fluorescent lipid, BODIPY FL-C_{12}, consisting of a green fluorophore linked to a 12-carbon fatty acyl tail. This molecule was previously used as a probe of membrane fluidity in a cyanobacterium (Sarcina *et al*., 2003). *E. coli* cells stained with BODIPY FL-C_{12} again show a fluorescent halo in confocal images (Fig. 2A). We sometimes observed gaps in staining close to sites of cell division (Fig. 2A), as previously observed with lipophilic cytoplasmic membrane stains (Fishov and Woldringh, 1999). The fluorescent halo is consistent with localization in the cytoplasmic membrane; however, at optical resolution it is not possible to directly distinguish the cytoplasmic (inner) and outer membranes in these cells. Using plasmolysis (Fishov and Woldringh, 1999) in cells elongated by growth in the presence of the cell division inhibitor cephalexin (Mullineaux *et al*., 2006) creates a large enough gap in the plasmolysis bays to permit fluorescence in the inner and outer membranes to be distinguished (Fishov and Woldringh, 1999). As previously observed (Fishov and Woldringh, 1999), plasmolysed cells do not show clear fluorescent halos (Fig. 2B), likely due to cytoplasmic shrinkage and cytoplasmic membrane infolding. No fluorescence could be detected in the outer cell layers in the plasmolysis bays, indicating that BODIPY FL-C_{12} localizes to the cytoplasmic membrane (Fig. 2B).

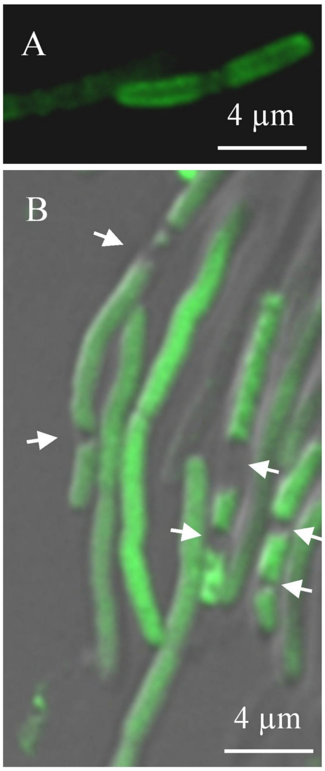

Fig. 2. BODIPY FL-C_{12} locates to the inner (cytoplasmic) membrane in *E. coli*.
A. Confocal fluorescence image of *E. coli* cells stained with BODIPY FL-C_{12}.
B. Superimposed Differential Interference Contrast (in grey-scale) and confocal fluorescence (in green) images for *E. coli* cells elongated by growth with cephalexin, stained with BODIPY FL-C_{12} and plasmolysed in 15% sucrose solution. The arrows indicate plasmolysis bays.

Measuring the lateral mobility of lipids and proteins using confocal FRAP

We used the techniques described and illustrated in Mullineaux *et al*. (2006) to measure diffusion coefficients for BODIPY FL-C_{12} and GFP-tagged plasma membrane proteins. Cells were elongated by growth in the presence of cephalexin, without detectable diffusion barriers along the length of the cell (Elowitz *et al*., 1999; Mullineaux *et al*., 2006). The use of elongated cells has two advantages. First, it increases the time required for fluorescence to re-equilibrate after bleaching, allowing more accurate determination of diffusion coefficients. In short cells, re-equilibration can be very rapid because of the limited diffusion space available to the fluorophore. Second, provided that the initial bleach is narrow compared to the length of the cell, it allows a simple data analysis in which complications due to cell poles can be ignored (Mullineaux *et al*., 1997). FRAP measurements were carried out using a wide confocal pinhole to give relatively low *z*-axis resolution, such that the two-dimensional fluorescence image sums information from the full volume of the cell. Note that this makes the resolution of the membrane halo less clear than in high-resolution images (Figs 1 and 2), which also have the benefit of a better signal-to-noise ratio because of slower image acquisition and signal averaging. The bleach was carried out by scanning a diffraction-limited line at high laser intensity across the short axis of the cell. Cells were then imaged at several time points after the bleach. Note that the initial bleach appears much broader than the diffraction-limited laser line, due to movement of the fluo-

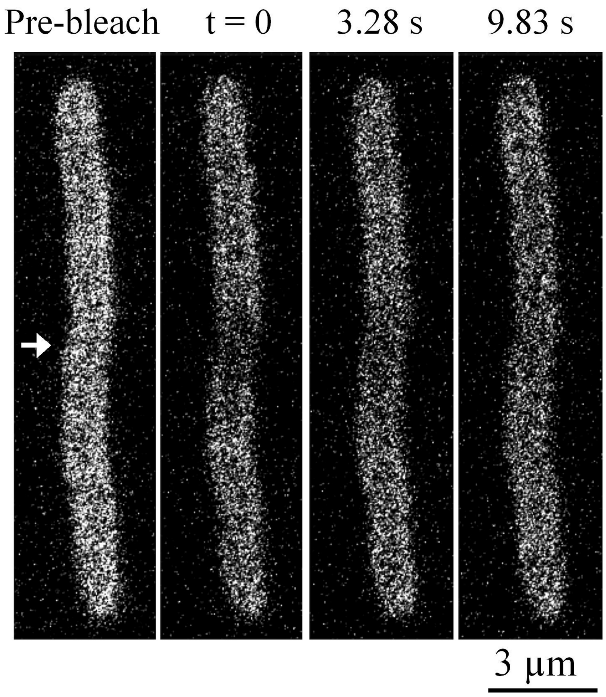

Fig. 3. FRAP image sequence for helix1021-GFP. 'Pre-bleach' shows an image prior to bleaching a line across the cell at the position indicated by the arrow. 't = 0' shows the first post-bleach image. Two subsequent time points from the image sequence are shown. Note the redistribution of fluorescence following the bleach.

rophore during the bleach and before acquisition of the first post-bleach image. The depth of the bleach that can be obtained is also decreased by movement of the fluorophore during the bleach. Neither effect precludes estimation of the diffusion coefficient, provided that fluorescence distribution is still in disequilibrium when the image sequence commences (Mullineaux *et al.*, 2006). One-dimensional profiles of fluorescence along the long-axis of the cell were extracted, and diffusion coefficients estimated using a previously published protocol that takes into account the initial measured width of the bleach and the time-dependence of fluorescence recovery at the bleach (Mullineaux *et al.*, 1997; 2006). A representative FRAP image sequence for helix1021-GFP is shown in Fig. 3, and full representative measurements with data analysis are shown in Supplementary Fig. S1 (helix1021-GFP) and Supplementary Fig. S2 (BODIPY FL-C_{12}).

Effect of temperature on the mobility of BODIPY FL-C_{12}

The green fluorescent fatty acid derivative BODIPY FL-C_{12} has previously been used as a probe of the fluidity of thylakoid membranes in cyanobacteria, where there is a pronounced dependence of its diffusion coefficient on temperature and the extent of fatty acid desaturation in the membrane (Sarcina *et al.*, 2003). We used confocal FRAP to measure the diffusion of BODIPY FL-C_{12} in the cytoplasmic membrane of *E. coli* as a function of temperature, for cells grown at 37°C. Supplementary Fig. S2 shows a representative measurement with data analysis. When cells were cooled we observed a step decrease in the lateral diffusion coefficient at about 28°C (Fig. 4). This resembles a classic phase transition from the liquid crystal to the crystalline gel states of the lipid bilayer, which in biological membranes usually occurs around 5–10°C below growth temperature (Hazel and Williams, 1990). However, as the *E. coli* cytoplasmic membrane is a very complex mixture of lipids and proteins, we cannot exclude the possibility that the change in BODIPY FL-C_{12} mobility is due to effects other than a simple lipid phase transition.

Cells generally acclimate to sustained growth at different temperatures by changing their membrane lipid composition, resulting in changes in fluidity and phase transition temperature (Thompson, 1989). Growth of *E. coli* at lower temperatures led to differences in the temperature dependence of BODIPY FL-C_{12} mobility (see Supplementary Fig. S3). Some effects of growth at different temperatures on the BODIPY FL-C_{12} diffusion coefficient are shown in Fig. 5A: note the higher BODIPY FL-C_{12} diffusion coefficient in cells grown at lower temperatures. At the extremes of the conditions tested, the diffusion coefficient of BODIPY FL-C_{12} differed by a factor of more than 4, ranging from 0.6 ± 0.2 $\mu m^2\ s^{-1}$ (mean ± SD) in cells grown at 37°C and measured at 17°C to 2.7 ± 0.5 $\mu m^2\ s^{-1}$ in cells grown and measured at 32°C (Figs 4 and 5).

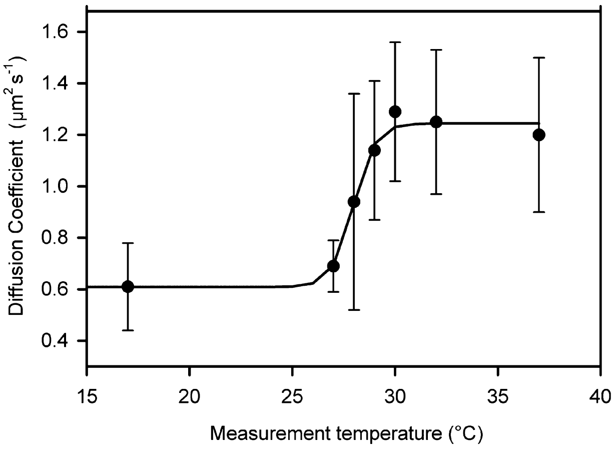

Fig. 4. Effect of temperature on the diffusion coefficient of BODIPY FL-C_{12}. *E. coli* DH5α cells grown at 37°C were labelled with the green fluorescent lipid analogue BODIPY FL-C_{12} in the cytoplasmic membrane. Cells were equilibrated to the temperatures shown before measuring the diffusion coefficient by FRAP as described in *Experimental procedures*. The mean diffusion coefficient at each temperature is shown (n = 10, ± SD). The sigmoidal fit indicates a phase transition melting temperature of 28°C.

© 2014 The Authors.

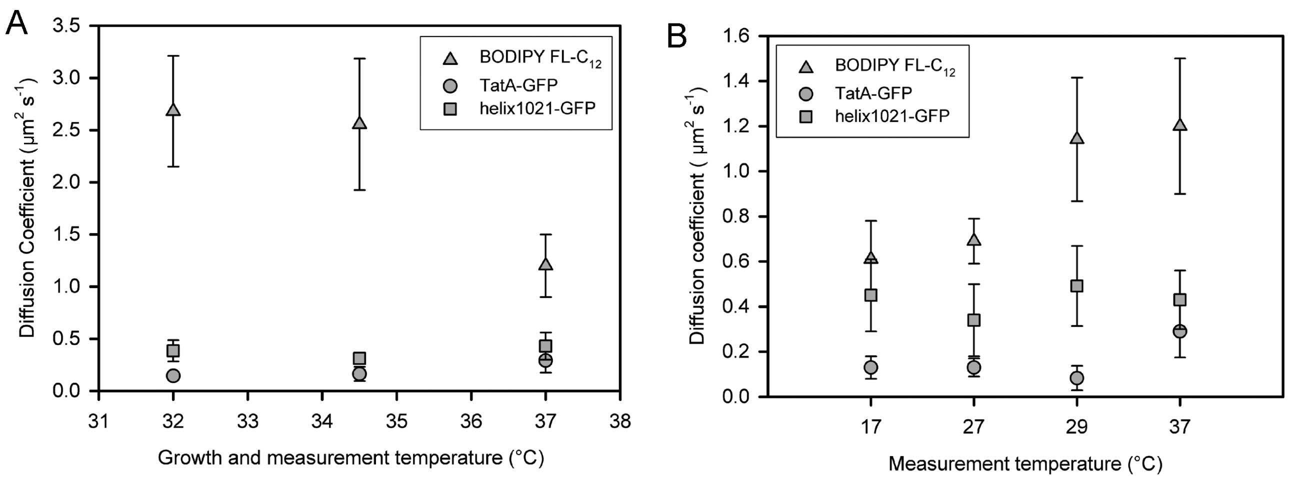

Fig. 5. Effect of growth and measurement temperatures on the lateral diffusion of lipids and proteins. Mean diffusion coefficients for BODIPY FL-C_{12} and two GFP-tagged membrane proteins are shown ($n = 10$, ± SD).
A. *E. coli* cells were grown at the temperatures indicated, and lateral diffusion coefficients measured by FRAP at the same growth temperature.
B. Cells were grown at 37°C and lateral diffusion coefficients measured at the temperatures indicated. Time required for sample preparation and equilibration at measurement temperature was about 20 min.

Effects of membrane protein expression and lipid biosynthesis mutations on the mobility of BODIPY FL-C_{12}

To further explore the factors controlling the mobility of BODIPY FL-C_{12}, we measured its diffusion coefficient in cells with different membrane protein and lipid compositions (summarized in Table 1). To perturb membrane protein composition, we used a mutant lacking the entire twin-arginine translocation (*tat*) operon (Wexler *et al.*, 2000), with and without a *pBad* plasmid overexpressing *tatABC* (Bolhuis *et al.*, 2001). After arabinose induction, the TatABC proteins pack the membrane (Bolhuis *et al.*, 2001). However, neither condition measurably perturbed the BODIPY FL-C_{12} diffusion coefficient (Table 1). We then also tried a series of mutants with deletions of genes coding for lipid biosynthesis enzymes (Dowhan, 1997): cardiolipin synthetase (*cls*); *pssA* (required for phosphatidylserine synthesis); *pgsA* (phosphatidylglycerophosphate synthetase) and phosphatidylserine decarboxylase (*psd*). Some of these mutants showed significant alteration of the BODIPY FL-C_{12} diffusion coefficient, by a factor of up to ~ 2 in either direction (Table 1).

Mobility of membrane proteins probed by FRAP

The same confocal FRAP techniques were used to measure lateral diffusion coefficients for two GFP-tagged

Table 1. Effects of mutations affecting membrane composition on protein and lipid mobility.

E. coli strain	Effect on membrane composition	BODIPY FL-C_{12} diffusion coefficient (μm^2 s^{-1}) ± SD	Helix1021-GFP diffusion coefficient (μm^2 s^{-1}) ± SD
Wild-type (DH5α)		1.2 ± 0.30	0.43 ± 1.3
Δ*tat*	Loss of twin-arginine translocon (Tat) subunits (Wexler *et al.*, 2000)	1.3 ± 0.58	0.32 ± 0.08
Δ*tat* overexpressing *tatABC*	Dense packing of the membrane with Tat subunits (Bolhuis *et al.*, 2001)	1.3 ± 0.25	
Δ*lpp*	Loss of lipoprotein Lpp	1.96 ± 0.71	0.38 ± 0.15
Δ*lpp* Δ*pgsA*	Loss of lipoprotein Lpp and reduced levels of phosphatidylglycerol and cardiolipin (Dowhan, 1997)	0.66 ± 0.22	0.43 ± 0.22
Δ*cls*	Loss of the major pathway for cardiolipin synthesis (Dowhan, 1997)	1.04 ± 0.41	0.50 ± 0.19
Δ*pssA*	Loss of phosphatidylserine and phosphatidylethanolamine (Dowhan, 1997)	2.6 ± 0.84	0.32 ± 0.07
Δ*psd*	Loss of phosphatidylethanolamine (Dowhan, 1997)	1.6 ± 0.53	0.52 ± 0.10

All cells were grown and measured at 37°C. Mean diffusion coefficients (± SD, $n = 10$) are shown.

© 2014 The Authors. *Molecular Microbiology* published by John Wiley & Sons Ltd.,

plasma membrane proteins; the simple model membrane protein helix1021-GFP and TatA-GFP, a tagged version of one of the three essential membrane-spanning proteins of the twin-arginine translocon (Tat) in *E. coli* (Ray *et al*., 2005). The mobility of TatA-GFP in the membrane has previously been investigated (Mullineaux *et al*., 2006; Leake *et al*., 2008). In both cases, the GFP tag is expected to be located on the cytoplasmic side of the membrane (Ray *et al*., 2005). Lateral diffusion coefficients were measured for helix1021-GFP and TatA-GFP at a range of measurement temperatures, for cells grown at different temperatures (Fig. 5). Both proteins diffused slower under all conditions than BODIPY FL-C_{12}, and under most conditions diffusion of TatA-GFP was significantly slower than helix1021-GFP (Fig. 5). More surprisingly, the mobility of the two proteins did not show the same temperature dependence as BODIPY FL-C_{12}, with no measurable change in mobility at the phase transition temperature (Fig. 5B). The standard deviations largely reflect real differences between individual cells, as the estimated errors in individual measurements are small. The mean diffusion coefficient of helix1021-GFP showed no significant temperature dependence over the range tested (one-way ANOVA, $P = 0.179$, $n = 10$). Pooling the entire set of data for different growth and measurement temperatures (Fig. 5), helix1021-GFP showed a mean diffusion coefficient (± SD) of $0.4 \pm 0.1\ \mu m^2\ s^{-1}$ ($n = 60$) with no significant differences under any of the conditions tested (one-way ANOVA, $P = 0.043$, $n = 10$).

We measured helix1021-GFP diffusion in the set of lipid biosynthesis mutants also used to measure effects of membrane lipid composition on BODIPY FL-C_{12} diffusion (Table 1). Effects on helix1021-GFP diffusion were generally much smaller than with BODIPY FL-C_{12} diffusion, and there was no obvious correlation between the effects on helix1021-GFP and BODIPY FL-C_{12} diffusion (Table 1). For example, the BODIPY FL-C_{12} diffusion coefficient was respectively increased and decreased by a factor of about 2 in the Δ*pssA* and Δ*lpp*Δ*pgsA* mutants, with no significant effects on helix1021-GFP diffusion (paired *t*-test, $P = 0.129$, $n = 10$ (Table 1).

The diffusion coefficient of TatA-GFP at different measuring temperatures (Fig. 5B) showed significant variation over the temperature range from 17°C to 37°C (one-way ANOVA, $P < 0.0005$, $n = 10$). However, there was no significant trend over the range 17–29°C (one-way ANOVA, $P = 0.088$, $n = 10$). Thus there is no significant change in mobility over the range where the lipid phase transition occurs (Fig. 5B). However, diffusion becomes significantly faster at 37°C (Tukey's test with 95% simultaneous confidence intervals). The mean diffusion coefficient for TatA-GFP over all the other conditions tested was $0.13 \pm 0.03\ \mu m^2\ s^{-1}$ ($n = 50$), which is consistent to within experimental error with values previously obtained from FRAP (Mullineaux *et al*., 2006) and single-particle tracking (Leake *et al*., 2008).

Video-rate TIRF microscopy of the helix1021-GFP model membrane protein

We further investigated the distribution and mobility of helix1021-GFP using video-rate TIRF microscopy, a technique previously used to quantify the behaviour of TatA in the *E. coli* cytoplasmic membrane (Leake *et al*., 2008). When expression of helix1021-GFP is strongly induced, it appears at optical resolution to be evenly distributed in the membrane (as seen by confocal microscopy in Fig. 1C). However, we found that when expression is weakly induced, GFP fluorescence was concentrated in distinct mobile foci in the membrane (typically one to two observed per cell), with no measurable localization bias to specific regions of the membrane around the cell. The foci could be observed most clearly by high-speed TIRF microscopy (Fig. 6A and Supplementary Movie). At higher protein concentrations these distinct spots of fluorescence were no longer detectable and the cellular fluorescence intensity increased. We applied automated single particle tracking to the motions of the spots to generate particle displacement trajectories, to quantify the intensity of the foci as a function of time after the start of the bleach, and to estimate the full width at half maximum (FWHM) of the intensity profile of each particle. Each displacement trajectory was converted into a mean-squared displacement (MSD) versus time interval trace (Leake *et al*., 2008), and an average MSD trace (Fig. 6B) was compiled from all automatically detected trajectories having a duration of at least 1 s (Robson *et al*., 2013). Individual MSD traces indicated a broad range of lateral diffusion coefficients from $0.05\ \mu m^2\ s^{-1}$ to $0.40\ \mu m^2\ s^{-1}$ with a mean value of $0.2 \pm 0.1\ \mu m^2\ s^{-1}$. Intensity analysis on the individual distinct foci was performed as described previously (Leake *et al*., 2006; 2008; Reyes-Lamothe *et al*., 2010; Badrinarayanan *et al*., 2012). In brief, this consisted of automated foci detection followed by iterative two-dimensional Gaussian fitting to determine the foci intensity centroid to a precision of a few tens of nm (Llorente-Garcia *et al*., 2014) followed by local background intensity subtraction in the immediate vicinity of each focus based upon an unbiased average pixel intensity estimate pixels in a square region of interest of area ~ 800 × 800 nm centred on each focus, excluding pixels from the circular two-dimensional point spread function image region of each focus within that square. The intensity analysis indicated a range of initial unbleached intensity values, with an average of 16 200 ± 6100 counts (± SD) on our camera detector. The estimated signal-to-noise ratio, with regard to the integrated foci pixel intensity signal and the local background pixel noise, was typically lower by a factor of ~ 2 (an

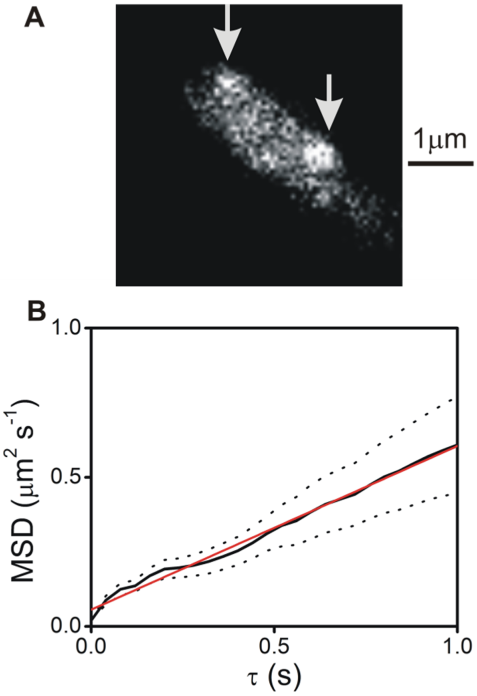

Fig. 6. TIRF imaging and single particle tracking.
A. TIRF image of *E. coli* cell expressing helix1021-GFP, with positions of two detected particles indicated (arrows). See also Movie S1.
B. Mean-squared displacement as a function of time interval τ taken from the average of nine individual tracked particle trajectories of 'long' duration of at least 1 s (solid line), with standard error bounds indicated (dotted lines), and linear fit (red), indicating an effective mean diffusion coefficient (± SD) of $0.14 \pm 0.02\ \mu m^2\ s^{-1}$ (as compared with a mean of ~ $0.2\ \mu m^2\ s^{-1}$ calculated using all trajectory data irrespective of duration length). Note this is marginally higher due to lower stoichiometry trajectories having a faster effective diffusion coefficient but photobleaching faster and thus lasting less than 1 s).

example of a pixel intensity profile of a focus in a live cell is given in Supplementary Fig. S4) than had been measured from previous studies involving a similar imaging set-up but investigating CytdB-GFP (Lenn *et al*., 2008) or TatA-YFP (Leake *et al*., 2008). However, this level of signal-to-noise ratio was consistent with other previous *in vivo* single-molecule fluorescence imaging studies using *E. coli* investigating lower stoichiometry molecular complexes (Leake, 2014) used in DNA replication (Reyes-Lamothe *et al*., 2010), DNA remodelling (Badrinarayanan *et al*., 2012), oxidative phosphorylation (Llorente-Garcia *et al*., 2014) and purified fluorescent protein (Plank *et al*., 2009). Prior calibration experiments using immobilized GFP on the surface of the glass coverslip indicated a unitary brightness of single GFP molecule of 670 ± 150 counts (± SD). We measured the penetration depth of the TIRF evanescent field as 110 ± 10 nm (± SD) and estimated previously that protein complexes embedded in the cytoplasmic membrane of *E. coli* were typically ~ 90 nm in height from the coverslip surface using the same cell immobilization protocol (Leake *et al*., 2006). This indicates that the number of GFP molecules associated with the distinct foci of helix1021-GFP is in the range of 30–70 molecules. At the lowest levels of expression used for the single-particle tracking TIRF microscopy data, we estimate ~ 180–840 helix1021-GFP molecules per cell, based on one to two foci observed per cell from TIRF images that are likely to encompass ~ 1/6 of the total cytoplasmic membrane area of the cell (Leake *et al*., 2006), whose stoichiometry is in the range ~ 30–70 molecules per focus. Measuring the intensity profile of the distinct foci indicated FWHM in the range 300–400 nm, 50–150 nm larger than measured from the surface-immobilized GFP molecules measured using the same imaging set-up (Plank *et al*., 2009; Reyes-Lamothe *et al*., 2010; Badrinarayanan *et al*., 2012; Llorente-Garcia *et al*., 2014). Therefore we can estimate that the real diameter of the foci is in the range 50–150 nm. Comparison of *E. coli* cell strains with a variety of different OXPHOS proteins (Llorente-Garcia *et al*., 2014) or components of the MukBEF DNA remodelling system (Badrinarayanan *et al*., 2012) labelled either with the standard Clontech enhanced GFP (as we use here), or with the 'definitively' monomeric fluorescent protein mCherry, yielded no statistically significant differences between stoichiometry distributions, with evidence of a unimodal population from surface immobilization assays of GFP (Plank *et al*., 2009). This indicates that putative GFP dimerization is unlikely to be an issue in our study.

Similar video-rate TIRF experiments using *E. coli* cells treated with BODIPY FL-C_{12} indicated a reasonably uniform fluorescence in the cell membrane with some putative low-level heterogeneity in the intensity but no distinct foci even at low concentrations of the dye.

Discussion

Rather than examining the diffusion of proteins in artificial membranes divorced from the native biological context, our approach here was to examine the diffusion of a simplified model protein in a real biological membrane, the cytoplasmic membrane of living *E. coli* cells, with the full native biological context intact. We created a fluorescent probe by expressing within the cell a fusion construct of a single membrane-spanning α-helix with a GFP reporter domain predicted to be exposed on the cytoplasmic side of the membrane. The protein localizes to the cytoplasmic membrane, and the GFP tag allows the use of FRAP to measure the lateral diffusion of the protein in the membrane, and to visualize the probe using video-rate, high-contrast TIRF microscopy. For comparison we also monitored the diffusion of BODIPY FL-C_{12}, a fluorescent lipid analogue with a 12-carbon fatty acid tail. In live *E. coli* cells this localizes preferentially to the plasma

membrane, so we are able to monitor diffusion of a model protein and a model lipid in the same membrane. The model lipid shows the expected temperature behaviour; for cells grown at 37°C its diffusion coefficient changes by a factor of about 2 over a narrow temperature range between 27°C and 30°C. This suggests a phase transition in the lipid (Hazel and Williams, 1990; Sarcina *et al.*, 2003) although the change in translational diffusion coefficient is small in comparison to that observed in simple lipid bilayers *in vitro*, where translational diffusion coefficients can change by orders of magnitude due to phase transition (Hazel and Williams, 1990).

Although the model membrane protein carries a relatively large GFP tag on the cytoplasmic side its diffusion is expected to be mainly controlled by the environment of the transmembrane domain, since the effective viscosity of the cell membrane is significantly greater than that of the cytoplasm. The diffusion coefficient of free GFP in the *E. coli* cytoplasm is typically 5–10 $\mu m^2\ s^{-1}$ (Elowitz *et al.*, 1999; Mullineaux *et al.*, 2006; Nenninger *et al.*, 2010), ~ 30 times faster than the diffusion coefficient we measured for the model membrane protein. In cells grown and measured at 37°C, the tagged protein diffuses roughly three times slower than the BODIPY label. The diffusion coefficient of a native membrane protein, GFP-tagged TatA, proved to be slower than the model protein under all the conditions that we tested. This could be explained by supramolecular interactions, including a tendency to homo-oligomerize (Leake *et al.*, 2008).

We examined the effect on protein diffusion of treatments that alter the effective viscosity of the membrane, as judged from the mobility of the BODIPY lipid probe. As compared to the standard situation (*E. coli* cells grown and measured at 37°C) the BODIPY diffusion coefficient can be decreased by a factor of about 2 by cooling below 27°C, or increased by a factor of about 2 by growing cells at lower temperatures, presumably due to changes in the lipid composition of the membrane (Thompson, 1989). However, none of these treatments had any significant effect on the diffusion coefficient of the model membrane protein. The BODIPY diffusion coefficient could be perturbed to a similar extent in mutants deficient in specific lipid biosynthesis enzymes, but again without concomitant effects on protein diffusion (Table 1).

The use of high-speed TIRF microscopy allowed us to observe a very inhomogeneous distribution of the protein in the membrane plane. When the model protein is weakly induced, and therefore present only at low concentrations in the membrane, GFP fluorescence was concentrated in mobile spots in the membrane. At higher protein concentrations these distinct spots of fluorescence were no longer detectable. This behaviour indicates that the formation of the fluorescent spots is not due to the concentration-dependent aggregation frequently observed when mem-

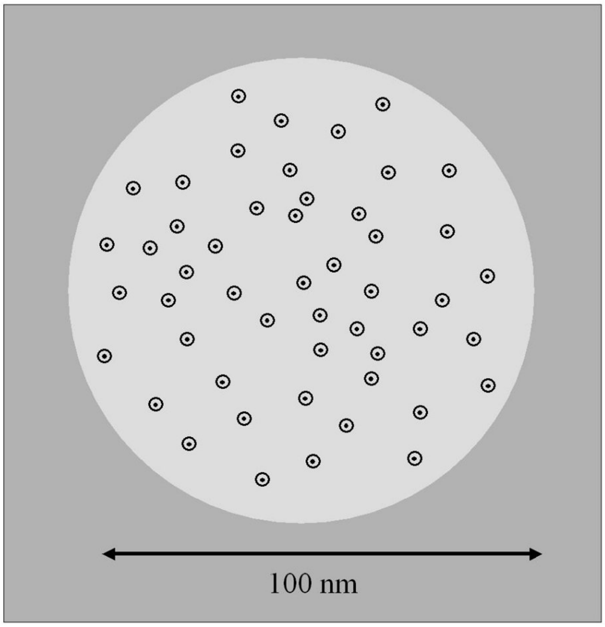

Fig. 7. Schematic model for the distribution of the helix1021-GFP protein construct in the membrane. Around 50 helix1021-GFP proteins are loosely concentrated in a zone of the membrane about 100 nm in diameter (shown as a lighter circle). The black spots indicate the approximate profile of the membrane-spanning α-helix, while the surrounding circles indicate the approximate profile of the GFP-tag.

brane proteins are overexpressed (Ray *et al.*, 2005). Rather, the protein has a tendency to segregate into domains of effective diameter of about 100 nm even at low concentrations. Each domain contains in the order of 50 helix1021-GFP proteins (Fig. 7). These domains are probably not observable at higher protein concentrations simply because they are too numerous to allow the spaces between them to be detected at optical resolution. We can estimate that the concentrated protein typically occupies less than 1% of a domain area (Fig. 7). Therefore it may well be undergoing faster micro-diffusion within each domain at timescales below our sampling time. The forces that hold these loose domains together are unclear and a topic for future study.

It is established that membrane proteins are surrounded by a shell of lipid molecules which is analogous to the solvent layer surrounding water-soluble proteins; several membrane proteins show greater affinity towards lipid in the liquid crystalline phase over that in the gel phase, consistent with packing considerations. For example, liquid crystalline phase lipid binding to Ca^{2+}-ATPase is ~ 25 times more likely than for gel phase lipid binding (Lee, 2003). As a working model for the behaviour of our model protein, we suggest that the protein has the ability to reorganize the surrounding lipid to such an extent that its diffusion is unaffected by changes in the

effective viscosity of the bulk lipid in the membrane. A preference of the protein for a particular lipid phase could also account for its tendency to segregate out into what appear to be loosely organized mobile domains in the membrane. An open question is whether our model protein is influencing the segregation of the membrane, or whether it is simply partitioning into microdomains that are already defined by specific interactions of the native lipids and proteins.

Several recent studies have examined the behaviour of native fluorescently tagged proteins in bacterial membranes (Johnson *et al.*, 2004; Ray *et al.*, 2005; Leake *et al.*, 2006; 2008; Lenn *et al.*, 2008; López and Kolter, 2010), and have observed a tendency for the protein to concentrate in mobile domains. These domains were characterized in some detail in the case of the cytochrome *bd* complex in *E. coli*, which is found in mobile domains on average approximately 100 nm in diameter, containing around 75 cytochrome *bd* complexes, and diffusing with a mean lateral diffusion coefficient of about 0.05 $\mu m^2 s^{-1}$ (Lenn *et al.*, 2008). Other OXPHOS complexes partition into separate domains which share similar characteristics (Llorente-Garcia *et al.*, 2014). These results already suggested that the plasma membrane of *E. coli* is a highly compartmentalized fluid. Our results here indicate that lateral segregation of the membrane can occur even with a very simple model membrane protein, and suggest that this may have a significant effect on the physical behaviour of the protein in the membrane. The precise factors that cause lateral segregation in the membrane, and the physiological consequences, are subjects for future study. It is likely that the observed robustness in membrane protein diffusion confers functional advantages in cells that are exposed to fluctuations in temperature.

Experimental procedures

Cell strains and molecular biology

All experiments were performed using the *E. coli* strain DH5α [*fhuA2* Δ*(argF-lacZ)U169 phoA glnV44 Φ80* Δ*(lacZ)M15 gyrA96 recA1 relA1 endA1 thi-1 hsdR17*]. The GFPmut3* (Cormack *et al.*, 1996) labelled membrane probes were expressed from the arabinose-inducible pBad24 vector (Guzman *et al.*, 1995). TatA-GFP was expressed from pBad24-$TatA_{GFP}BC_s$ (Ray *et al.*, 2005). For the helix1021-GFP construct the truncated version of *sll1021* was amplified via PCR using genomic DNA from *Synechocystis* sp. PCC 6803 creating an EcoRI site at the 5′ end and a *gfp* overlap on the 3′ end. The fluorescent tag was amplified from pJDT1 (Thomas *et al.*, 2001) creating a *sll1021* overlap at the 5′ end and a HindIII site at the 3′ end. To fuse the two PCR products an overlap extension PCR (Shevchuk *et al.*, 2004) was performed using external primer and the end-product cloned into pBad24 via EcoRI and HindIII and transformed into DH5α. Restriction enzymes were FastDigest® (Fermentas). PCR was with PfuUltra II Fusion HS DNA Polymerase (Stratagene). Ligations used the Quick Ligase Kit from NEB. For primer sequences see Supplementary Table S1.

The lipid biosynthesis deletion mutants were created using the λ Red and FLP recombinase system (Datsenko and Wanner, 2000). Kanamycin (Δ*pgsA*, Δ*cls*, Δ*pssA*, Δ*psd*) and chloramphenicol (Δ*lpp*) resistant cassettes were amplified via PCR from pKD13 or pKD3 respectively. They were inserted into the *E. coli* genome to replace corresponding genes. The correct insertion was confirmed by PCR and the stability of the deletions was verified several times during the period of the experiments.

Preparation of cells for confocal FRAP measurements

Bacterial cultures were grown aerobically overnight in Luria–Bertani (LB) medium (Sambrook and Russell, 2001) at 37°C under constant shaking (180 r.p.m.) and supplemented with ampicillin (50 $\mu g\ ml^{-1}$) where needed. For FRAP measurements, overnight cultures were diluted 1:100 and long non-septated cells were produced by adding cephalexin to a final concentration of 30 $\mu g\ ml^{-1}$ (Ishihara *et al.*, 1983) and grown for up to 120 min at the different temperatures under constant shaking to mid-exponential phase (OD_{600} = 0.3–0.4). Wild-type cultures were grown to mid-exponential phase with 1 μM BODIPY FL-C_{12} (Invitrogen) and washed several times in fresh LB. For induction of the GFP-tagged membrane proteins, L-arabinose was added to the diluted culture [500 μM for TatA-GFP and 133 mM (2%) for helix1021-GFP] and cells were grown at the appropriate temperature to mid-exponential phase. For transfer to the microscope drops of cell culture were spotted onto LB-agar plates and allowed to adsorb onto the agar surface. Small blocks of agar holding adsorbed cells were cut from the plate and placed in a laboratory-built sample holder connected to a temperature-controlled circulating water-bath, covered with a coverslip and placed under the microscope (Mullineaux *et al.*, 2006). Sample preparation and temperature equilibration took about 20 min in total.

Confocal imaging and FRAP data analysis

FRAP measurements and data analysis were carried out according to Mullineaux *et al.* (2006) using a Nikon PCM2000 laser-scanning confocal microscope equipped with an Argon laser run at 100 mW. A wide (50 μm) confocal pinhole was used to reduce the *z*-axis resolution and thus collect information from the full depth of the cell. Excitation at 488nm was reduced by a factor of 32 via neutral-density filters for imaging, ensuring that repeat imaging did not measurably bleach the sample. The filters were manually lifted (for about 1 s) for the high laser power bleach. For the bleach across the short axis of the elongated cell the *x*-scanning mode was selected. Pre- and post-bleach scans were recorded separately at 1.64 s intervals over a 512 by 512 pixel area using the *xy* mode for scanning. The measurement area was either 29 μm or 58 μm square. Fluorescence emission was detected between 500 and 527 nm. Image analysis used Image-Pro 6.2 (Media Cybernetics). Pre- and post-bleach image sequences were combined and a one-dimensional fluores-

cence profile along the long axis of the cell was extracted as a line-profile summing data widthways across the cell for the entire sequence. In SigmaPlot 10.0 (Jandel Scientific) the diffusion coefficient D was obtained by fitting to a one-dimensional diffusion equation (Mullineaux *et al.*, 1997; 2006). SigmaPlot was also used to perform Student's *t*-tests and for fitting of sigmoidal curves to plots of D versus temperature. Further statistical analyses were made using MINITAB.14 (Minitab). We conducted one-way ANOVAS followed by Tukey's tests to assess significant differences, considering a *P* value of < 0.005 as significant. High-resolution fluorescence and DIC images were recorded using a Leica TCS SP5 laser-scanning confocal microscope with excitation at 488 nm and emission at 500–520 nm. A 6× line-average was used, and the confocal pinhole was set to give a *z*-axis resolution of about 0.8 μm.

Preparation of cells for TIRF microscopy

Cells were sub-cultured into M63-glucose medium (Atlas, 1993) with 1mM arabinose for induction of helix1021-GFP expression, then grown with shaking at 30°C for 3 h. The cell suspension was injected through a poly-L-lysine-coated tunnel slide (Leake *et al.*, 2006) and left to incubate for 20 min. Excess medium was flushed through the tunnel slide to remove unbound cells.

TIRF microscopy and single particle tracking

A home-built inverted TIRF microscope with 473 nm excitation wavelength was used with specifications as described (Leake *et al*., 2006; 2008; Lenn *et al.*, 2008). Fluorescence emission was imaged at 50 nm/pixel in frame-transfer mode at 25 Hz by a 512 × 512-pixel, cooled, back-thinned electron-multiplying charge-coupled device camera (iXon+ DV885-BI, Andor Technology). Images were sampled for ~ 10 s at room temperature (20°C). Fluorescent particles were automatically detected and tracked on each consecutive image frame using automated custom-written image-analysis software and a mean-squared displacement (MSD) calculated for each particle trajectory, with an estimate for lateral diffusion coefficient generated using a linear fit to the MSD versus time interval relation (Leake *et al.*, 2008).

Cell fractionation and SDS-PAGE with Western blotting

Spheroplasts were produced according to Randall and Hardy (1986) by lysing the cell wall with lysozyme and stabilizing spheroplasts with $MgSO_4$. They were harvested by gentle centrifugation and the supernatant was collected: this corresponds to the soluble fraction from the periplasm. The periplasmic fraction was centrifuged a second time to ensure removal of all spheroplasts. After an additional wash step, spheroplasts were fractioned into a membrane and soluble cytoplasmic fractions according to Thomas *et al.* (2001). Spheroplasts were burst by sonication and the membranes were collected by ultracentrifugation (250 000 *g* for 30 min). The supernatant corresponded to the cytoplasmic fraction. The membrane fraction was washed once in buffer and, after a second centrifugation step, resuspended in buffer. The proteins of the membrane fraction were solubilized overnight by adding a small droplet of Triton X-100 to the buffer. Proteins were separated by 15% SDS-PAGE, semi-dry electroblotted onto Hybond-R polyvinylidene difluoride (PVDF) membrane (GE Healthcare) and probed with antibodies against GFP (Invitrogen). A horseradish peroxidase-conjugated anti-mouse IgG secondary antibody and ECL enhanced chemiluminescence detection kit (GE Healthcare) permitted visualization of GFP.

Acknowledgements

The research was supported by a Biotechnology and Biological Sciences Research Council grant (BB/E009571) to C.W.M., by the Oxford Centre for Integrative Systems Biology (OCISB) through the Biotechnology and Biological Sciences Research Council (Q.X.), the Engineering and Physical Science Research Council (A.R., M.C.L.), the Royal Society (M.C.L.) and Hertford College Oxford (M.C.L.). The authors declare no conflict of interest.

References

Aisenbrey, C., and Bechinger, B. (2004) Investigations of polypeptide rotational diffusion in aligned membranes by ^{2}H and ^{15}N solid-state NMR spectroscopy. *J Am Chem Soc* **126:** 16676–16683.

Atlas, R.M. (1993) *Handbook of Microbiological Media*. Boca Raton, FL, USA: CRC Press.

Badrinarayanan, A., Reyes-Lamothe, R., Uphoff, S., Leake, M.C., and Sherratt, D.J. (2012) *In vivo* architecture and action of bacterial structural maintenance of chromosome proteins. *Science* **338:** 528–531.

Bolhuis, A., Mathers, J.E., Thomas, J.D., Barrett, C., and Robinson, C. (2001) TatB and TatC form a functional and structural unit of the twin-arginine translocase from *Escherichia coli*. *J Biol Chem* **276:** 20213–20219.

Cormack, B.P., Valdivia, R.H., and Falkow, S. (1996) FACS-optimized mutants of the green fluorescent protein (GFP). *Gene* **173:** 33–38.

Datsenko, K.A., and Wanner, B.L. (2000) One-step inactivation of chromosomal genes in *Escherichia coli* K-12 using PCR products. *Proc Natl Acad Sci USA* **97:** 6640–6645.

Denich, T.J., Beaudette, L.A., Lee, H., and Trevors, J.T. (2003) Effect of selected environmental and physico-chemical factors on bacterial cytoplasmic membranes. *J Microbiol Methods* **52:** 149–182.

Dowhan, W. (1997) Molecular basis for membrane phospholipid diversity: why are there so many lipids? *Annu Rev Biochem* **66:** 199–232.

Elowitz, M.B., Surette, M.G., Wolf, P.E., Stock, J.B., and Leibler, S. (1999) Protein mobility in the cytoplasm of *Escherichia coli*. *J Bacteriol* **181:** 197–203.

Engelman, D.M. (2005) Membranes are more mosaic than fluid. *Nature* **438:** 578–580.

Fishov, I., and Woldringh, C.L. (1999) Visualization of membrane domains in *Escherichia coli*. *Mol Microbiol* **32:** 1166–1172.

Frye, L.D., and Edidin, M. (1970) The rapid intermixing of cell

surface antigens after formation of mouse-human heterokaryons. *J Cell Sci* **7:** 319–335.
Fulbright, R.M., Axelrod, D., Dunham, W.R., and Marcelo, C.L. (1997) Fatty acid alteration and the lateral diffusion of lipids in the plasma membrane of keratinocytes. *Exp Cell Res* **233:** 128–134.
Guzman, L.-M., Belin, D., Carson, M.J., and Beckwith, J. (1995) Tight regulation, modulation, and high-level expression by vectors containing the arabinose P-BAD promoter. *J Bacteriol* **177:** 4121–4130.
Hazel, R.J., and Williams, E.E. (1990) The role of alteration in membrane lipid composition in enabling physiological adaptation of organisms to their physical environment. *Prog Lipid Res* **29:** 167–227.
Huang, F., Parmryd, I., Nilsson, F., Persson, A.L., Pakrasi, H.B., Andersson, B., and Norling, B. (2002) Proteomics of *Synechocystis* sp. strain PCC6803. Identification of plasma membrane proteins. *Mol Cell Proteomics* **1:** 956–966.
Hunte, C. (2005) Specific protein–lipid interactions in membrane proteins. *Biochem Soc Trans* **33:** 938–942.
Ishihara, A., Segall, J.E., Block, S.M., and Berg, H.C. (1983) Coordination of flagella on filamentous cells of *Escherichia coli*. *J Bacteriol* **155:** 228–237.
Jacobson, K., Mouritsen, O.G., and Anderson, R.G.W. (2007) Lipid rafts: at a crossroad between cell biology and physics. *Nat Cell Biol* **9:** 7–14.
Jin, A.J., Edidin, M., Nossal, R., and Gershfeld, N.L. (1999) A singular state of membrane lipids at cell growth temperatures. *Biochemistry* **38:** 13275–13278.
Johnson, A.S., van Horck, S., and Lewis, P.J. (2004) Dynamic localization of membrane proteins in *Bacillus subtilis*. *Microbiology* **150:** 2815–2824.
Leake, M.C. (2014) Analytical tools for single-molecule fluorescence imaging *in cellulo*. *Phys Chem Chem Phys*. doi:0.1039/C4CP00219A
Leake, M.C., Chandler, J.H., Wadhams, G.H., Bai, F., Berry, R.M., and Armitage, J.P. (2006) Stoichiometry and turnover in single, functioning membrane protein complexes. *Nature* **443:** 355–358.
Leake, M.C., Greene, N.P., Godun, R.M., Granjon, T., Buchanan, G., Chen, S., *et al*. (2008) Variable stoichiometry of the TatA component of the twin-arginine protein transport system observed by *in vivo* single-molecule imaging. *Proc Natl Acad Sci USA* **105:** 15376–15381.
Lee, A.G. (2003) Lipid–protein interactions in biological membranes: a structural perspective. *Biochim Biophys Acta* **1612:** 1–40.
Lenn, T., Leake, M.C., and Mullineaux, C.W. (2008) Clustering and dynamics of cytochrome bd-I complexes in the *Escherichia coli* plasma membrane *in vivo*. *Mol Microbiol* **70:** 1397–1407.
Lindblom, G., Orädd, G., Rilfors, L., and Morein, S. (2002) Regulation of lipid composition in *Acholoeplasma laidlawii* and *Escherichia coli* membranes: NMR studies of lipid lateral diffusion at different growth temperatures. *Biochemistry* **41:** 11512–11515.
Llorente-Garcia, I., Lenn, T., Erhardt, H., Harriman, O., Liu, L.-N., Robson, A., *et al*. (2014) Single-molecule *in vivo* imaging of bacterial respiratory complexes indicates delocalized oxidative phosphorylation. *Biochim Biophys Acta* **1837:** 811–824.
López, D., and Kolter, R. (2010) Functional microdomains in bacterial membranes. *Genes Dev* **24:** 1893–1902.
Matsumoto, K., Kusaka, J., Nishibori, A., and Hara, H. (2006) Lipid domains in bacterial membranes. *Mol Microbiol* **61:** 1110–1117.
Mileykovskaya, E., and Dowhan, W. (2009) Cardiolipin domains in prokaryotes and eukaryotes. *Biochim Biophys Acta* **1778:** 2084–2091.
Morein, S., Andersson, A., Rilfors, L., and Lindblom, G. (1996) Wild-type *Escherichia coli* cells regulate the membrane lipid composition in a 'window' between gel and non-lamellar structures. *J Biol Chem* **271:** 6801–6809.
Mullineaux, C.W., Tobin, M.J., and Jones, G.R. (1997) Mobility of photosynthetic complexes in thylakoid membranes. *Nature* **390:** 421–424.
Mullineaux, C.W., Nenninger, A., Ray, N., and Robinson, C. (2006) Diffusion of green fluorescent protein in three cell environments in *Escherichia coli*. *J Bacteriol* **188:** 3442–3448.
Murata, N. (1989) Low-temperature effects on cyanobacterial membranes. *J Bioenerg Biomemb* **21:** 61–75.
Natale, P., Bruser, T., and Driessen, A.J.M. (2008) Sec- and Tat-mediated protein secretion across the bacterial cytoplasmic membrane – distinct translocases and mechanisms. *Biochim Biophys Acta* **1778:** 1735–1756.
Nenninger, A., Mastroianni, G., and Mullineaux, C.W. (2010) Size dependence of protein diffusion in the cytoplasm of *Escherichia coli*. *J Bacteriol* **192:** 4535–4540.
Plank, M., Wadhams, G.H., and Leake, M.C. (2009) Millisecond timescale slimfield imaging and automated quantification of single fluorescent protein molecules for use in probing complex biological processes. *Integr Biol (Camb)* **1:** 602–612.
Randall, L.L., and Hardy, L.S. (1986) Correlation of competence for export with lack of tertiary structures of the mature species: a study *in vivo* of maltose-binding in *E. coli*. *Cell* **46:** 921–928.
Ray, N., Nenninger, A., Mullineaux, C.W., and Robinson, C. (2005) Location and mobility of twin-arginine translocase subunits in the *Escherichia coli* plasma membrane. *J Biol Chem* **280:** 17961–17968.
Reyes-Lamothe, R., Sherratt, D.J., and Leake, M.C. (2010) Stoichiometry and architecture of active DNA replication machinery in *Escherichia coli*. *Science* **328:** 498–501.
Rilfors, L., and Lindblom, G. (2002) Regulation of lipid composition in biological membranes – biophysical studies of lipids and lipid synthesizing enzymes. *Colloids Surf B Biointerfaces* **26:** 112–124.
Robson, A., Burrage, K., and Leake, M.C. (2013) Inferring diffusion in single live cells at the single molecule level. *Philos Trans R Soc Lond B Biol Sci* **368:** 20130029.
Sambrook, J., and Russell, D.W. (2001) *Molecular Cloning: A Laboratory Manual*. Plainview, NY, USA: Cold Spring Harbor Laboratory Press.
Sarcina, M., Murata, N., Tobin, M.J., and Mullineaux, C.W. (2003) Lipid diffusion in the thylakoid membranes of the cyanobacterium *Synechococcus* sp.: effects of fatty acid desaturation. *FEBS Lett* **553:** 295–298.
Shevchuk, N.A., Bryksin, A.V., Nusinovich, Y.A., Cabello, F.C., Sutherland, M., and Ladisch, S. (2004) Construction of long DNA molecules using long PCR-based fusion of

several fragments simultaneously. *Nucleic Acids Res* **32:** e19.

Singer, S.J., and Nicolson, G.L. (1972) The fluid mosaic model of the structure of cell membranes. *Science* **175:** 720–731.

Szalontai, B., Nishiyama, Y., Gombos, Z., and Murata, N. (2000) Membrane dynamics as seen by Fourier transform infrared spectroscopy in a cyanobacterium, *Synechocystis* PCC 6803 – the effects of lipid unsaturation and the protein-to-lipid ratio. *Biochim Biophys Acta* **1509:** 409–419.

Thomas, J.D., Daniel, R.A., Errington, J., and Robinson, C. (2001) Export of active green fluorescent protein to the periplasm by the twin-arginine translocase (Tat) pathway in *Escherichia coli*. *Mol Microbiol* **39:** 47–53.

Thompson, G.A. (1989) Membrane acclimation by unicellular organisms in response to temperature change. *J Bioenerg Biomembr* **21:** 43–60.

Tominaga, M., Hashimoto, S., and Nakashima, N. (2004) Effect of phase transition on the electrochemical behavior of ferredoxin embedded in an artificial lipid membrane film. *J Electroanal Chem* **561:** 13–20.

Vereb, G., Szöllősi, J., Matkó, J., Nagy, P., Farkas, T., Vigh, L., *et al*. (2003) Dynamic, yet structured: the cell membrane three decades after the Singer-Nicolson model. *Proc Natl Acad Sci USA* **100:** 8053–8058.

Wexler, M., Sargent, F., Jack, R.L., Stanley, N.R., Bogsch, E.G., Robinson, C., *et al*. (2000) TatD is a cytoplasmic protein with DNase activity. *J Biol Chem* **275:** 16717–16722.

Supporting information

Additional supporting information may be found in the online version of this article at the publisher's web-site.

© 2014 The Authors. *Molecular Microbiology* published by John Wiley & Sons Ltd.,
